# ^18^F-JK-PSMA-7 PET/CT for staging intermediate- or high-risk prostate cancer patients before radical prostatectomy: a pilot study

**DOI:** 10.1186/s41824-022-00161-2

**Published:** 2023-01-23

**Authors:** Irina Vierasu, Gaetan Van Simaeys, Nicola Trotta, Simon Lacroix, Guy Bormans, Simone Albisinni, Thierry Quackels, Thierry Roumeguère, Serge Goldman

**Affiliations:** 1 Nuclear Medicine Department & PET/Biomedical Cyclotron Unit, Hôpital Erasme, Université libre de Bruxelles (ULB), Route de Lennik 808, 1070 Brussels, Belgium; 2 Center for Microscopy and Molecular Imaging (CMMI), Université libre de Bruxelles (ULB), Charleroi, Gosselies, Belgium; 3 Radiopharmaceutical Recherche, KU Leuven, Leuven, Belgium; 4 Urology Department, Cliniques Universitaires de Bruxelles, HUB, Hôpital Erasme, Université libre de Bruxelles (ULB), Brussels, Belgium

**Keywords:** High-risk prostate cancer, Intermediate-risk prostate cancer, Initial staging, Pre-surgery ^18^F-JK-PSMA7 PET/CT, Post-surgery ^18^F-JK-PSMA7 PET/CT

## Abstract

**Background:**

Positron emission tomography/computed tomography (PET/CT) using radiotracers that bind to the prostate-specific membrane antigen (PSMA) is mainly used in biochemical recurring prostate cancer. The aim of our study was to assess the usefulness of ^18^F-JK-PSMA-7 PET/CT for local and nodal staging in patients with intermediate- and high-risk prostate cancer (PCa) prior to radical prostatectomy, as compared to conventional imaging techniques.

**Methods:**

We enrolled a total of 10 patients with intermediate- and high-risk PCa diagnosed by multiparametric-MRI followed by systematic and targeted biopsies, eligible for radical prostatectomy with extended lymph node dissection. Clinical team was blind to the results of the pre-surgery ^18^F-JK-PSMA-7 PET/CT at times of clinical decision and surgery. One month post-surgery, 18F-JK-PSMA-7 PET/CT was repeated and the results of both scans were unblinded. A third ^18^F-JK-PSMA-7 PET/CT could be acquired at a later time point depending on PSA progression.

**Results:**

All pre-surgery ^18^F-JK-PSMA-7 PET/CT was positive in the prostatic region, while MRI was negative in the prostate in one patient. We also detected positive pelvic lymph nodes in two patients (one high-risk, one intermediate-risk PCa) on pre-surgery and post-surgery ^18^F-JK-PSMA-7 PET/CT. No positive pelvic lymph nodes were reported on pre-surgical CT and MRI. ^18^F-JK-PSMA-7 PET/CT detected bladder involvement in one patient and seminal vesicles involvement in two patients; this malignant extension was undetected by the conventional imaging techniques. SUVmax in prostate lesions had an average value of 11.51 (range 6.90–21.49). SUVmean in prostate lesions had an average value of 7.59 (range 5.26–14.02).

**Conclusion:**

This pilot study indicates that pre-surgery ^18^F-JK-PSMA-7 PET/CT provides valuable information in intermediate- and high-risk PCa, for surgery planning with curative intent.

## Introduction

Positron emission tomography/computed tomography (PET/CT) using radiotracers that bind to the prostate-specific membrane antigen (PSMA) is mainly used in biochemical recurrence (BCR) of prostate cancer (PCa). A variety of PET tracers targeting PSMA have been developed and proved their value in this indication. Considering the clinical and logistical advantages of ^18^F, over the initially used ^68^Ga labelling, the new class of ^18^F-PSMA has attracted a strong interest among the nuclear medicine community. Among these new tracers, ^18^F-JK-PSMA-7 has been extensively studied by Jülich/Köln group, which conducted several clinical trials proving its efficacy for the detection of active lesions in PCa patients with biochemical recurrence (Hohberg et al. 2019; Dietlein et al. 2019; Dietlein et al. 2020). We conducted a clinical study that confirmed ^18^F-JK-PSMA-7 PET/CT as a robust imaging method in PCa patients with biochemical recurrence, even at low PSA values (Vierasu et al. 2022).

While the most recent guidelines of the European Association of Urology (EAU) support the use of PSMA PET/CT imaging for biochemical recurrence of PCa after radical prostatectomy, they remain elusive regarding its use in primary staging (Mottet et al. 2022). The guidelines of the European Society of Nuclear Medicine published in 2021 suggest that PSMA PET/CT might be helpful for the initial staging prior to radical treatment in high-risk PCa (Ceci et al. 2021). The pro-PSMA study published by Hofman and Lawrentschuk (2020) showed that ^68^Ga-PSMA-11 PET-CT provides superior accuracy and can replace CT and bone scanning in the staging of men with high-risk prostate cancer.

The present trial is a pilot study applying the new ^18^F-JK-PSMA-7 for the evaluation of patients with intermediate- and high-risk PCa prior to radical prostatectomy. The method was compared to the conventional imaging techniques for the detection of local and lymph node extension of the disease that may impact on the therapeutic decision.

## Materials and methods

### Patients

Patients’ recruitment began in September 2021 and ended in April 2022.

Patients with intermediate- and high-risk prostate cancer according to the European guidelines were included (Mottet et al. 2022). They had been diagnosed by multiparametric-MRI and systematic and targeted biopsies and were eligible for radical prostatectomy with extended lymph node dissection. Patients with previously treated prostate cancer were excluded.

**Fig. 1:**
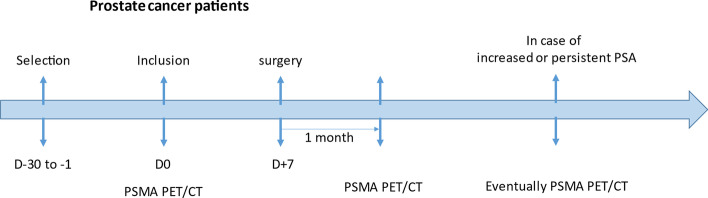
Schematic representation of the study

After diagnostic and staging with conventional imaging techniques according to guidelines, we performed an initial ^18^F-JK-PSMA7 PET/CT (see Fig. 1). The multidisciplinary oncology committee remained blinded to PSMA PET/CT results when discussing the findings of standard radiological imaging on which a surgical decision was taken. Similarly, the surgeons were blinded to the pre-surgery PSMA PET/CT results during the intervention. One month post-surgery, a second ^18^F-JK-PSMA-7 PET/CT scan was performed and the results of the first and second scans were then disclosed. Biological testing with PSA assessment was performed.

Patients’ follow-up included clinical and biological evaluations at 1, 3, 6 and 12 months post-surgery. A third ^18^F-JK-PSMA-7 PET/CT could be acquired during follow-up, depending on PSA progression.

### ^18^F-JK-PSMA-7 PET/CT Imaging

^18^F-JK-PSMA-7 was produced by the Radiopharmacy of the UZ Leuven (KULeuven) in accordance with the appropriate cGMP standard for clinical PET materials (manufacturing authorization number 1771 IMP).

All PET/CT scans were performed on a Philips Vereos digital PET/CT (Philips Medical Systems, Cleveland, Ohio, USA), the characteristics of which have been described previously (Zhang et al. 2018). PET acquisitions were performed 140 min after an intravenous injection of 4 MBq/kg of ^18^F-JK-PSMA-7 radioactivity (range 277–396 MBq).

We did not perform dynamic acquisitions, so we have no data to provide on the evolution of TBR over time. We based our imaging protocol (acquisition at 140 min) on the data published by the Jülich/Köln group, using the same tracer (Hohberg et al. 2019). In one of the papers published by this group, a 50% TBR increase is shown between 100 and 140 min post-injection.

The average dose of radioactivity injected throughout the study was 343.88 ± 2.39 (mean ± SD) for a mean body weight of 85.4 kg (range 69–100 kg).

Fifteen minutes after radiotracer injection, patients received furosemide intravenously for forced diuresis to avoid penetration of urine with concentrated radioactivity into the permeable epithelial surface of the bladder and to reduce focal ureteral, renal and bladder activity.

Whole-body PET acquisition was performed in all patients, from the skull vertex to the feet. It lasted approximately 14 min for a total of 17 bed positions (1 min per bed position for 10 bed positions from the skull to mid-thigh and 30 s per bed position for the 7 bed positions from thighs to feet). PET acquisitions were combined with a low-dose non-contrast-enhanced CT (50 mAs, 120 kV). Photon attenuation correction was performed from the CT images.

PET images were reconstructed using the 3D ordered subset expectation maximization (OSEM) algorithm implemented on the Philips Vereos PET/CT system and set to 3 iterations, 15 subsets and a 3D Gaussian filter with a full width at half*-*maximum of 6 mm (Vierasu et al. 2022).

### Image analysis

A licensed nuclear medicine specialist with 17 years of experience (IV) analysed the images by visual inspection. SUV measurements were obtained on each lesion identified on the PET images.

The anatomical localization of positive lymph nodes identified on pre-surgery ^18^F-JK-PSMA-7 PET/CT was compared with those identified on pre-biopsy abdominal-pelvic MRI and those mentioned in the final histological report.

Pre-surgical and post-surgical ^18^F-JK-PSMA-7 PET/CT were compared. Lymph nodes that were present after lymphadenectomy following standard procedures were recorded.

The PSMA uptake in the prostate was mainly focal. In the patient 01, the PSMA uptake in the prostate was extended rather nodular.

We followed the anatomical areas for the definition of the extend of lymph nodes dissection as described by Fossati et al.: I = obturator nodes; II = external iliac nodes; III = internal iliac nodes; IV = common iliac nodes; and V = presacral nodes (Fossati et al. 2017).

## Results

Twelve patients were initially included in the study, but two of them were excluded: one ultimately refused surgery and one patient developed bone metastases after inclusion leaving a final population of ten men.

At the pre-surgical evaluation, all patients had negative thoraco-abdominal CT. Five patients performed bone scan and five patients performed NaF PET/CT that were all considered negative. The 10 patients included in our study had a mean age of 66 years (range 57–72).

All patients underwent robotic-assisted laparoscopic prostatectomy with extended lymph node dissection by two experienced surgeons (TQ and SA). Lymph node dissection template followed EAU guidelines, including ileo-obturator, external iliac, hypogastric and common iliac lymph nodes until the ureter crossing.

PSA value (mean ± SD) was 11.27 ± 5.71 before surgery.

Table 1 summarizes the PIRADS and Gleason scores, ISUP staging and decision after surgery. Five patients had PIRADS 5 prostate lesions, and four patients had PIRADS 4 prostate lesions. In one patient, there was no morphological lesion on prostate MRI.

**Table 1:** PIRADS prostate lesions classification, Gleason scores, ISUP staging and decision after surgery

Patient	MRI lesion classification	Gleason scores		ISUP		Decision after surgery
		Before surgery	After surgery	Before surgery	After surgery	
P01	PIRADS 5	9 (4 + 5)	9 (4 + 5)	5	5	Radiotherapy of prostatic region and of the pelvic lymph nodes areas; long-term hormonotherapy
P02	PIRADS 5	7 (4 + 3)	9 (4 + 5)	3	5	Radiotherapy of prostatic region and of the pelvic lymph nodes areas; long-term hormonotherapy
P04	PIRADS 4	9 (5 + 4)	9 (5 + 4)	5	5	Hormonotherapy
P05	PIRADS 4	7 (3 + 4)	7 (4 + 3)	2	3	Follow-up
P06	PIRADS 5	7 (3 + 4)	7 (3 + 4)	2	2	Biological recurrence: salvage radiotherapy of the prostatic and pelvic regions and hormonotherapy
P07	PIRADS 4	7 (3 + 4)	7 (3 + 4)	2	2	Follow-up
P08	no lesion	7 (3 + 4)	7 (4 + 3)	2	3	Follow-up
P09	PIRADS 5	7 (3 + 4)	7 (3 + 4)	2	2	Follow-up
P11	PIRADS 4	8 (4 + 4)	8 (4 + 4)	4	4	Follow-up
P12	PIRADS 5	8 (4 + 4)	8 (4 + 4)	4	4	Follow-up

Before surgery, two patients were considered ISUP5 (Gleason 9 = 4 + 5), two patients were considered ISUP4 (Gleason 8 = 4 + 4), one patient was considered ISUP3 (Gleason 7 = 4 + 3) and 5 patients were considered ISUP2 (Gleason 7 = 3 + 4). On final pathology report, the patient who was initially considered as ISUP3 was upgraded to ISUP5 and two of the patients who were initially ISUP2 were reclassified as ISUP3.

The histopathological findings before and after surgery are shown in Table 2. Six patients had a ductal component, seven patients exhibited a cribriform pattern, and seven patients showed capsular involvement. The pathologist did not find any neuroendocrine differentiation. PSA values (mean ± SD) were 0.47 ± 0.99 and 0.04 ± 0.08, one month and 3 months post-surgery, respectively. The PSA values at one month post-surgery for patients P01 and P02 were 1.2 ng/ml and 3.1 ng/ml, respectively.

**Table 2:** Histopathology before and after surgery

Patient	Ductal component		Cribriform pattern		Neuroendocrine differentiation		Capsular involvement	
	Before surgery	After surgery	Before surgery	After surgery	Before surgery	After surgery	Before surgery	After surgery
P01	−	+	+	+	−	−	−	+
P02	−	+	−	+	−	−	+	+
P04	+	+	+	+	−	−	−	−
P05	+	+	−	+	−	−	−	+
P06	−	+	+	+	−	−	−	+
P07	−	−	−	−	−	−	−	+
P08	−	−	−	−	−	−	−	+
P09	−	−	−	−	−	−	−	−
P11	−	−	+	+	−	−	−	−
P12	+	+	+	+	−	−	−	+

All ten patients had a positive pre-surgery ^18^F-JK-PSMA-7 PET/CT (see Table 3).

**Table 3:** Summary of results

Regions	PSMA before surgery		PSMA after surgery	
	**+**	**−**	**+**	**−**
All regions	10	0	3	7
Prostatic region	10	0	2	8
Pelvic lymph nodes	2	8	2	8

On the pre-surgery ^18^F-JK-PSMA-7 PET/CT, all patients had PSMA-positive lesions in the prostate region, but two patients also had PSMA-positive pelvic lymph nodes.

The pre-surgery PSMA PET/CT of patient P01 showed five PSMA-positive pelvic lymph nodes, one in the presacral area (area V), two in the right pelvis near the bladder (area I) and two right internal iliac nodes (area III). In this patient, there were also PSMA-positive abnormalities in the wall of the urinary bladder, confirmed by post-surgical histological findings.

In the patient with no morphological MRI lesion, the pre-surgery ^18^F-JK-PSMA-7 PET/CT showed PSMA-positive abnormalities in both prostate lobes.

Table 4 shows the localizations of prostate lesions, pelvic lymph nodes and seminal vesicles involvement on preoperative PSMA PET/CT, while Table 5 shows the corresponding information on MRI. The average SUVmax value for the prostate lesions was 11.51, and the average SUVmean was 7.59. In pelvic lymph nodes, the average SUVmax and SUVmean were 17.44 and 12.66, respectively.

**Table 4:** Pre-surgery PSMA PET/CT prostate lesions localization, seminal vesicles, bladder and pelvic lymph nodes involvement

Patient	Prostate lobes	Prostate sectors	Seminal vesicles	Pelvic lymph nodes	Bladder lesion
P01	Both	2p, 1p, 3a, 3p, 4a, 9a, 6a, 6p, 5a, 11p	Right	Positive	Positive
P02	Both	7p, 4p, 3p, 9p, 9a, 6p, 5p	Both	Positive	Negative
P04	Both	4a, 3a, 9a, 10a, 11a, 12a	Negative	Negative	Negative
P05	Right	2p	Negative	Negative	Negative
P06	Left	7p, 9p	Negative	Negative	Negative
P07	Both	6p, 3p, 9p	Negative	Negative	Negative
P08	Both	1a, 7a, 3a, 9a	Negative	Negative	Negative
P09	Both	5p, 11p	Negative	Negative	Negative
P11	Left	11p, 12p, 11a, 9p, 10p, 9a, 7a	Negative	Negative	Negative
P12	Right	3p, 4p	Negative	Negative	Negative

**Table 5:** Pre-surgery MRI prostate lesions localization, seminal vesicles and pelvic lymph nodes involvement

Patient	Prostate lobes	Prostate sectors	Seminal vesicles	Pelvic lymph nodes
P01	Right	4a, 5a	Negative	Initially deemed negative but positive after reviewing
P02	Both	4p, 3p, 9p, 5p	Negative	Negative
P04	Left	11a, 12a	Negative	Negative
P05	Right	2p	Negative	Negative
P06	Left	7p, 9p	Negative	Negative
P07	Both	6p, 3p, 9p	Negative	Negative
P08	No lesion	-	Negative	Negative
P09	Both	5p, 11p	Negative	Negative
P11	Left	11p, 12p, 11a, 9p, 10p, 9a, 7a	Negative	Negative
P12	Right	3p, 4p	Negative	Negative

The SUVmax of lesions in the bladder of patient P01 was 11.79, and the SUVmean was 7.68.

In two patients, there were two bone abnormalities with low PSMA expression that were considered negative by the nuclear medicine specialist. One bone lesion had a SUVmax of 2.9, and the second one had a SUVmax of 3.1. The aspect was similar on the post-surgery PSMA PET/CT.

We performed the same quantitative analyses as reported by the Jülich/Köln group (Hohberg et al. 2019). The gluteus maximus muscle (SUVmean) served as a reference region for the calculation of tumour-to-background ratios (TBRs). Blood radioactivity concentration was measured in the lumen of the left ventricle of the heart for all sequentially acquired PET images.

On the pre-surgery PSMA PET/CT, the SUVmean in the left gluteal muscles was 0.35 ± 0.06. The SUVmean in left cardiac ventricle was 1.34 ± 0.13. On the post-surgery PSMA PET/CT, the SUVmean in the muscle and in the left cardiac ventricle were 0.32 ± 0.06 and 1.09 ± 0.06, respectively.

On the post-surgery ^18^F-JK-PSMA-7 PET/CT, three patients had positive lesions (P01, P02 and P04).

P01 patient had residual PSMA-positive lesions in prostatic bed, and all the pelvic lymph nodes visualized on the pre-surgery ^18^F-JK-PSMA-7 PET/CT were found again. We were not able to assess PSMA-positive abnormalities in the urinary bladder on the post-surgery ^18^F-JK-PSMA-7 PET/CT because of a high urinary activity level. Incidentally, for the post-surgery ^18^F-JK-PSMA-7 PET/CT the patient did not have a urinary catheterization, although he had had one for the pre-surgery scan.

In patient P01, the SUVmax value of the residual prostatic lesion was 27 and the SUVmean of this lesion was 8.79. The SUVmax in the residual lymph nodes was 18.38, and the SUVmean was 3.93.

Patient P02 had a positive post-surgery PSMA PET/CT in pelvic lymph nodes already shown on the pre-surgery PSMA PET/CT.

The SUVmax of these lymph nodes was 10.26.

Here is the brief description of the patients 01 and 02.

The patient P01, 57 years old, had a PSA of 6.5 ng/ml and a cT2 staging at the digital rectal examination. Thirteen out of fourteen biopsies showed a bilateral acinar adenocarcinoma with cribriform differentiation and perineural sheathing, Gleason score 9 (5 + 4), up to 90% pattern 5, ISUP grade 5 and no neuroendocrine differentiation.

**Fig. 2:**
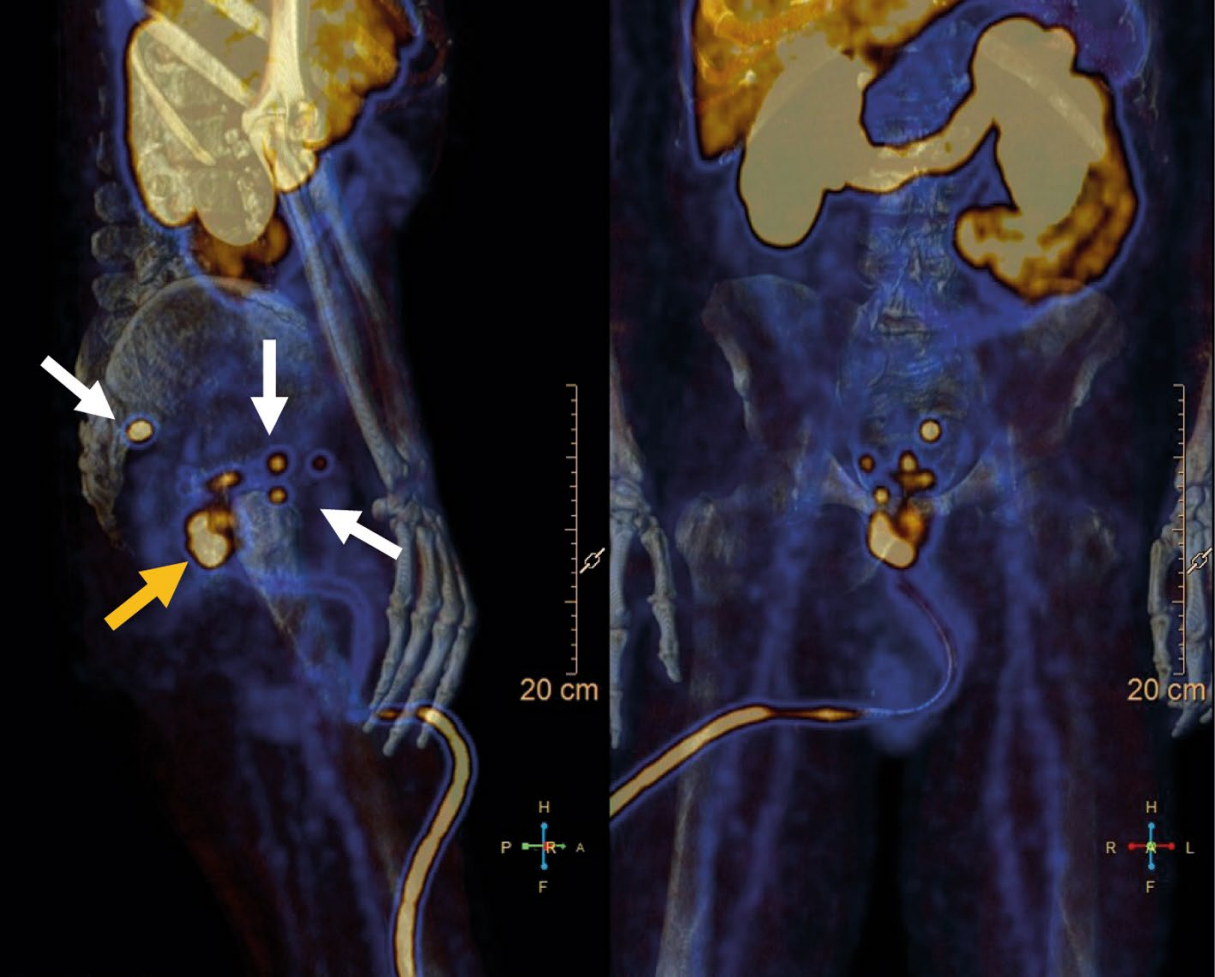
Patient 01 pre-surgery PSMA PET/CT: the white arrows represent the pelvic lymph nodes; the yellow arrow represents the prostate lesion

**Fig. 3:**
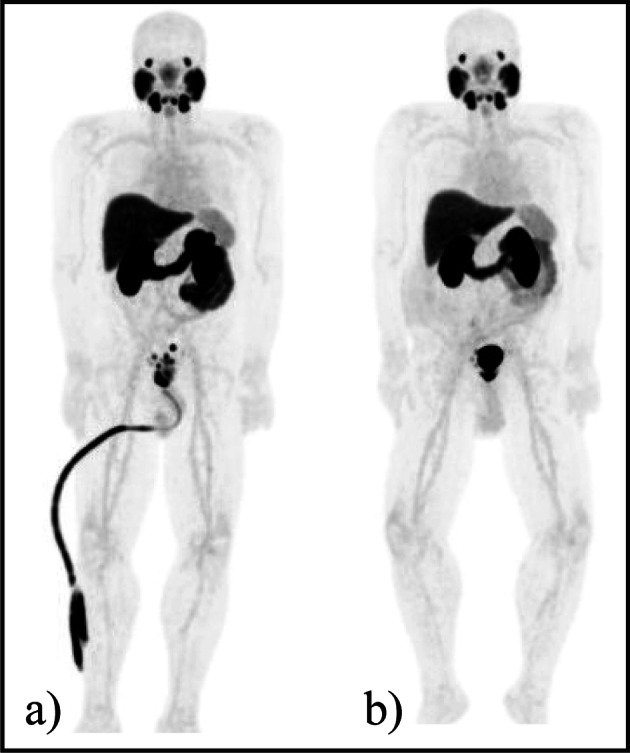
**a** Patient 01 MIP pre-surgery PSMA PET/CT; **b** Patient 01 MIP post-surgery PET/CT

**Fig. 4:**
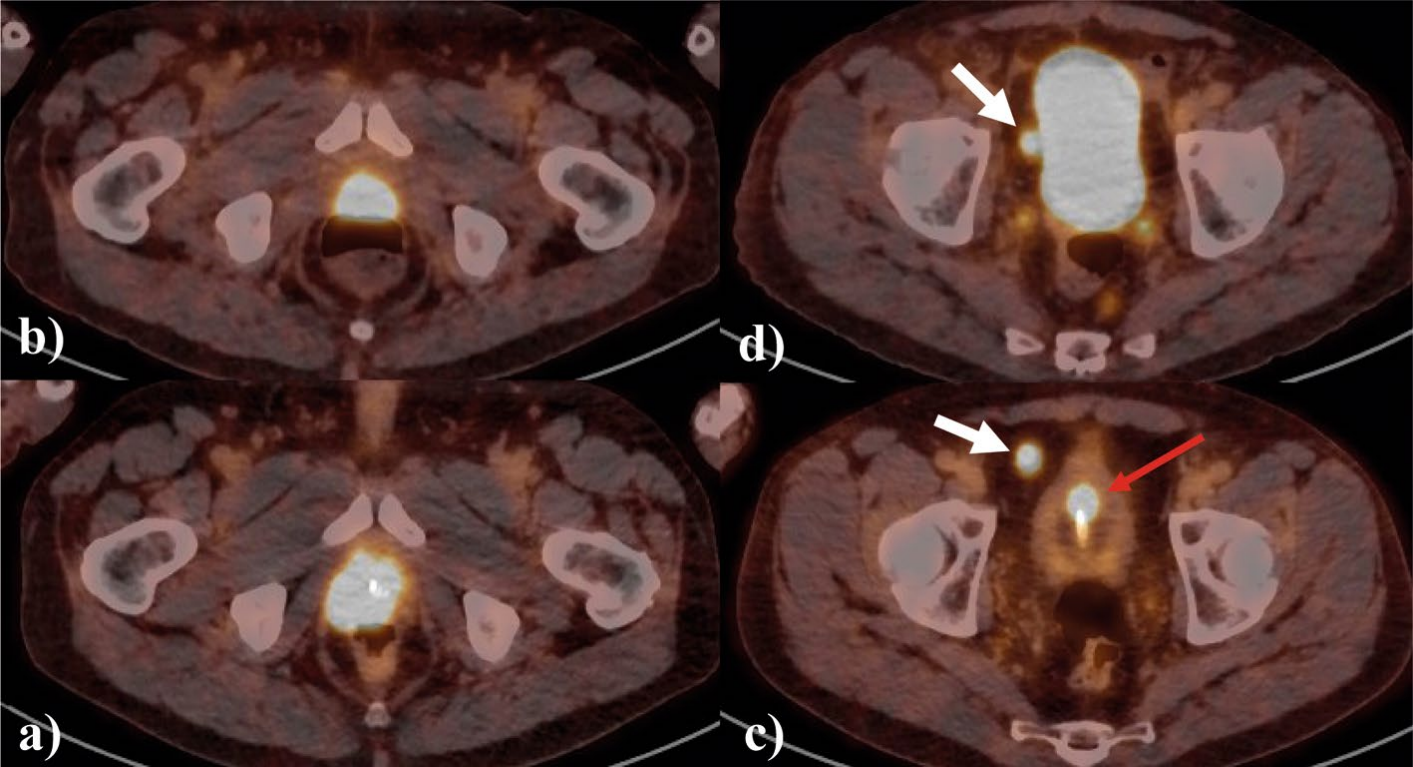
Patient 01 **a** prostate lesion on pre-surgery PSMA PET/CT; **b** prostate bed residual lesion on post-surgery PSMA PET/CT; **c** para-vesical lymph node on pre-surgery PSMA PET/CT; **d** para-vesical lymph node on post-surgery PSMA PET/CT (white arrow); red arrow represents a lesion in urinary bladder wall

The pre-surgery PSMA PET/CT showed the presence of extensive PSMA over-expressing abnormalities in both prostatic lobes and in contact with the rectal wall: 2p, 1p, 3a, 3p, 4a, 4p, 9a, 9p, 6a, 6p, 5a, 5p, 11p. Also, there were lymph nodes over-expressing PSMA: one left presacral node, zone V; two nodes in the right para-vesical area, zone I and 2 nodes in the right internal iliac area, zone III. The pre-surgery and post-surgery PSMA PET/CT images are shown in Figs. 2, 3 and 4.

This patient presented with PSMA-positive abnormalities in the urinary bladder wall despite a negative CT scan. The fact that this patient underwent urinary catheterization during pre-surgery ^18^F-JK-PSMA-7 PET/CT helped us to identify these PSMA-positive lesions.

The post-surgery PSMA PET/CT showed residual PSMA-positive abnormalities in the prostatic bed and the persistence of pelvic PSMA-positive lymph nodes as described on the pre-surgery PSMA PET/CT. The evaluation of the previously described abnormalities in the urinary bladder wall was not possible, in the absence of urinary catheterization.

The patient P01 had an extended radiotherapy in the entire prostatic bed and pelvic region with boost on the PSMA-positive lymph nodes associated with a long-term hormonotherapy.

The patient 02 had an initial PSA of 21 ng/ml and presented a bilateral acinar-type prostate adenocarcinoma, with twelve out of fourteen positive biopsies, capsule invaded without extraprostatic tumour extension; Gleason score 7 (4 + 3), ISUP grade 3.

MRI showed two foci PIRADS 5/5 in the posterolateral and posteromedial part of the right middle peripheral zone (sectors 4p, 3p, 9p) and paramedian of the right apical peripheral zone (5p). CT and PET/CT NaF were considered negative. The abnormality of the lateral arch of the 5th right rib was considered not suspicious for metastasis.

The final histopathological analysis showed a Gleason 4 + 5 = 9, presence of cribriform architecture, ISUP grade 5, stage pT3bN0R0.

**Fig. 5:**
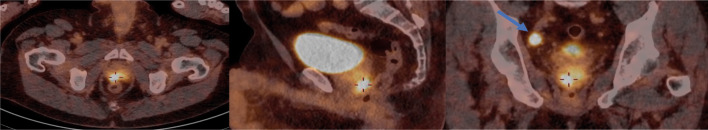
PSMA-positive prostate lesion on the pre-surgery PSMA PET/CT of the patient 02; the blue arrow represents an iliac lymph node

The pre-surgery PSMA PET/CT showed abnormalities over-expressing PSMA in both prostatic lobes and in a presacral lymph node, zone V and a right external iliac lymph node, zone II (Fig. 5).

Very low-expressing PSMA abnormality in the lateral arch of the right 5th rib was considered not metastatic.

The post-surgery PSMA PET/CT showed the same two lymph nodes described above, and a stability of low-expressing PSMA abnormality in the 5th rib.

Post-surgery PSA level was 3.1 ng/ml. The patient was treated by radiotherapy in the entire prostatic bed and pelvic region associated with a long-term hormonotherapy. At 3-month follow-up, the PSA was 0.01 ng/ml.

Patient P04 had residual PSMA-positive abnormalities in the prostate region, with a SUVmax value of 6.93 and a SUVmean of 3.4.

The decisions of the multidisciplinary oncology committee after surgery are presented in Table 1. In two patients with PSMA-positive pelvic lymph nodes after surgery, the treatment was radiotherapy of the prostate region and pelvic lymph nodes areas, together with long-term hormonotherapy. In six patients, the decision was medical follow-up. One patient received hormonotherapy only. One patient had a biological recurrence with a PSA value of 0.13 ng/ml at one month post-surgery, and 0.24 ng/ml at 3 months post-surgery. He was treated with salvage radiotherapy and 6 months of hormonotherapy.

## Discussion

This prospective pilot study showed that in selected patients, especially with high-risk PCa, ^18^F-JK-PSMA-7 PET/CT prior to radical prostatectomy could be useful to evaluate lymph node involvement, but also to identify other malignant lesions as in the case of the patient P01 who presented with PSMA-positive abnormalities in the urinary bladder wall despite a negative CT scan. The fact that this patient underwent urinary catheterization during pre-surgery ^18^F-JK-PSMA-7 PET/CT helped us to identify these PSMA-positive lesions. Therefore, urinary catheterization could be useful to visualize lesions that over-express PSMA in the urinary bladder.

Regarding lymph node involvement, we noted that ^18^F-JK-PSMA-7 PET/CT revealed more lesions than MRI or CT.

A recent paper showed that the patients in whom pelvic lymph node metastases were suspected on preoperative PSMA imaging had a significantly increased risk of biochemical disease progression after surgery (Meijer et al. 2022).

The additional lymph nodes detected on ^18^F-JK-PSMA-7 PET/CT were generally small in diameter which precluded their detection based on their size on morphological imaging. Detection of those lesions by ^18^F-JK-PSMA-7 PET/CT is likely the result of two factors: high overexpression of PSMA and high resolution of PET imaging using ^18^F-based tracers that ensured better signal recovery than more energetic isotopes such as ^68^Ga (Hoffmann et al. 2022; Dietlein et al. 2015). These combined factors probably allowed high SUVmax values in these small lesions (22.62 in patient P01 and 12.26 in patient P02).

These high SUVmax values might represent important biomarkers highly associated with conventional prognostic factors such as ISUP or lymph node status as recently suggested also by Bodar et al. (Bodar et al. 2022).

Our study is consistent with the multi-centric pro-PSMA trial published in March 2020, which showed that PET-CT with ^68^Ga-PSMA-11 provides superior accuracy compared to conventional imaging (CT and bone scan) and affects disease management in high-risk patients (Hofman and Lawrentschuk 2020).

Regarding the unspecific bone abnormalities, similar aspects were already described in the literature as potential pitfalls (Grünig et al. 2021; Ayati et al. 2022). In our clinical practice, we also noticed these bone aspects with a tendency of upstaging the disease especially when the images are analysed by less experienced nuclear medicine physicians. The undetectable PSA values at 1 and 3 months after surgery in both patients with these bone abnormalities confirmed their benign nature.

The positive pre-surgery PSMA PET/CT could have been useful in guiding lymph nodes dissection during surgery, which mean adaptation of the therapeutic strategy. The patient P01 had an extended radiotherapy in the entire prostate and pelvic region with boost on the PSMA-positive lymph nodes associated with a long-term hormonotherapy. BRCA 1/2 testing and neuroendocrine component research were negative in this young patient (57 year).

The PSMA-positive lymph nodes on the pre-surgery PSMA PET/CT in patient P02 could also have guided the lymph nodes dissection during surgery. This patient had an extended radiotherapy of the prostate region with boost on the PSMA-positive lymph nodes and long-term hormonotherapy.

The concept of PSMA-guided surgery already described for the lymph node dissection might be developed and helpful for reducing recurrence risk in intermediate- or high-risk PCa (Maurer et al. 2020; Luining et al. 2022). However, a recent article draws attention to the fact a negative pre-surgery PSMA PET/CT does not rule out lymph node metastases and, consequently, extended pelvic lymph node dissection remains the gold standard for primary nodal staging despite the potential post-surgical complications (Luining et al. 2022).

The limitations of this pilot study are the small number of patients and the mixed population of both intermediate- and high-risk patients. Therefore, it should get confirmation in a larger and homogenous population of patients.

## Conclusions

This pilot study indicates that the new ^18^F-JK-PSMA-7 tracer in pre-surgical setting is a promising tool for the initial staging of patients with high-risk and intermediate-risk PCa. It appears to have a high sensitivity for the detection of invaded lymph nodes and the detection of prostate and peri-prostatic lesions. It deserves further prospective evaluation in larger populations of patients, to determine the clinical impact of present findings in the management of the disease and obviously the guidance of prostate biopsy in MRI-negative cases.

## Data Availability

The original contributions presented in the study are included in the article/supplementary materiel, and further inquiries can be directed to the corresponding author.

## Abbreviations

PCa: Prostate cancer
PSMA: Prostate-specific membrane antigen
PET/CT: Positron emission tomography/computed tomography
